# Effects of Prenatal Exposure to a Low-Dose of Bisphenol A on Sex Differences in Emotional Behavior and Central Alpha_2_-Adrenergic Receptor Binding

**DOI:** 10.3390/ijms21093269

**Published:** 2020-05-05

**Authors:** Davide Ponzi, Laura Gioiosa, Stefano Parmigiani, Paola Palanza

**Affiliations:** 1 Department of Medicine and Surgery, University of Parma, 43121 Parma, Italy; laura.gioiosa@unipr.it (L.G.); paola.palanza@unipr.it (P.P.); 2 Department of Chemistry, Life Sciences and Environmental Sustainability, University of Parma, 43121 Parma, Italy; stefano.parmigiani@unipr.it

**Keywords:** bipshenol A, estrogen, alpha_2_ adrenergic receptors, sex differences, catecholamines, ethology

## Abstract

Prenatal exposure to bisphenol A (BPA) influences the development of sex differences neurologically and behaviorally across many species of vertebrates. These effects are a consequence of BPA’s estrogenic activity and its ability to act as an endocrine disrupter even, at very low doses. When exposure to BPA occurs during critical periods of development, it can interfere with the normal activity of sex steroids, impacting the fate of neurons, neural connectivity and the development of brain regions sensitive to steroid activity. Among the most sensitive behavioral targets of BPA action are behaviors that are characterized by a sexual dimorphism, especially emotion and anxiety related behaviors, such as the amount of time spent investigating a novel environment, locomotive activity and arousal. Moreover, in some species of rodents, BPA exposure affected males’ sexual behaviors. Interestingly, these behaviors are at least in part modulated by the catecholaminergic system, which has been reported to be a target of BPA action. In the present study we investigated the influence of prenatal exposure of mice to a very low single dose of BPA on emotional and sexual behaviors and on the density and binding characteristics of alpha_2_ adrenergic receptors. Alpha_2_ adrenergic receptors are widespread in the central nervous system and they can act as autoreceptors, inhibiting the release of noradrenaline and other neurotransmitters from presynaptic terminals. BPA exposure disrupted sex differences in behavioral responses to a novel environment, but did not affect male mice sexual behavior. Importantly, BPA exposure caused a change in the binding affinity of alpha_2_ adrenergic receptors in the locus coeruleus and medial preoptic area (mPOA) and it eliminated the sexual dimorphism in the density of the receptors in the mPOA.

## 1. Introduction

Bisphenol A (BPA) is an environmentally ubiquitous monomer used in the synthesis of polycarbonate plastics and epoxy resins that are employed in the manufacturing of food and beverage containers and dental sealants [1]. BPA is found in measurable amounts in human tissues, organs and biological fluids, such as blood, placenta and breast milk [2,3,4] levels that are not due to assay or laboratory contamination, but from every-day environmental exposure [5], implying that human fetuses and infants are at risk of exposure. Owing to its ability to bind intracellular and membrane-associated estrogen receptors, BPA acts similarly to endogenous estrogen leading to the activation of genomic and nongenomic mechanisms [6] that, during period of early development and plasticity, have long term effects on several neuroendocrine systems [7,8] and behaviors [9]. In this regard, abundant experimental data show that BPA can interfere with the development of the central nervous system at doses below its US reference dose of 50 ug/kg/day and within the estimated daily intake for adults and children [10].

Among the most sensitive endpoints of an early exposure to BPA are those systems and traits that will develop to become sexually dimorphic in the adult. Sexual dimorphism is the result of a complex interaction between the sex complement (the differences within males’ and females’ cells in the expression of genes associated with sex chromosomes) and the organizational role that sex steroids play during critical periods of development [11]. Perinatal exposure to sex steroids exerts a potent, long term influence on the developing nervous system. These effects result in permanent sex differences involving the number and size of neurons, synaptic formation, dendritic length and distribution patterns of serotoninergic, dopaminergic and noradrenergic systems [12,13]. Through its aromatization to estradiol, in rodents testosterone causes masculinization and defeminization of males’ brain structures and of behaviors under their control [12].

Amid the first areas of the CNS that were found to be sexually dimorphic are regions regulating reproductive behaviors, such as the medial preoptic area (mPOA) and the anteroventral periventricular nucleus (AVPV) of the hypothalamus [14]. However, differences in the size of brain nuclei, number of cells and connectivity have been found in other regions of the brain not directly involved with reproductive behaviors, such as the hippocampus [15], the locus coeruleus (LC) [16], the amygdala and other limbic regions [14,17]. The LC is a noradrenergic nucleus of the brainstem that is involved in arousal, vigilance, attention, cognition and the stress response [18,19,20]. In the rat, the perinatal surge of testosterone induces a clear sex difference in the volume of the adult’s LC, with females’ LC that is larger [16,21]. In mice, a similar but transient sexual difference in the number of tyrosine hydroxylase (TH; the rate limiting enzyme for dopamine and noradrenaline) neurons is observed and possibly mediated by estrogen-β receptors [22]. Perinatal exposure to low doses of BPA reduced the sex differences in LC volume in rats [23,24] and mice [25]: in these studies BPA also eliminated sex differences in exploratory behaviors and locomotor activity in the open field (OF), consistently with previous studies from our laboratory [26,27]. Such a behavioral response to a novel environment reflects increased arousal and is assumed to be, at least in part, modulated by the noradrenergic system.

The medial preoptic area (mPOA) of the hypothalamus is a sexually dimorphic area that regulates sex behaviors in males [28,29] and maternal behavior [30]. This area is composed of several sexually dimorphic subregions, such as the sexually dimorphic nucleus (SDN) of the POA, which is larger in males [31] and the AVPV, larger in females [32]. The mPOA is rich in estrogen receptors [14], in adrenergic receptors and innervation originating from the LC [33] and in neurons expressing TH [14]. In this regard, a sexually dimorphic population of neurons expressing TH which is dependent on ERalpha receptors has been reported [34]. Catecholaminergic innervation of the mPOA is necessary for the luteinizing hormone (LH) surge that precedes ovulation [35], and for the expression of lordosis, in female rats [36], and for males’ sexual behavior, possibly through a mechanism that involves the binding of dopamine to alpha_2_ adrenergic receptors [37,38]. The sexual dimorphism in these hypothalamic areas was reduced by early exposure to low doses of BPA [39,40]. Likewise, a similar exposure paradigm to BPA affected sex specific behaviors such as maternal care in mice [41] and behaviors related to reproduction in rats [42,43].

Based on our previously reported sex-dependent effects of prenatal exposure to BPA on behavioral responses mediated by the catecholaminergic system of the house mouse [44], in the present study we investigated whether the exposure to the same low dose of BPA (10 µg/Kg BW) had long term, sex dependent disruptive effects on the noradrenergic system. This dose is below the US reference dose of 50 ug/kg/day, but above the EU tolerable daily intake of 4 ug/kg/day (http://www.efsa.europa.eu/en/topics/topic/bisphenol). First, we investigated the effects of this exposure regime on sex differences in reproductive and emotional behaviors that are, at least in part, modulated by the catecholaminergic system. Then, we explored the possible functional link between behavior and neurobiology by studying the density and affinity of alpha_2_-adrenergic receptor in the LC and mPOA, two sexually dimorphic areas associated with anxiety and reproductive behaviors. Alpha_2_-adrenergic receptors are transmembrane proteins that belong to the monoaminergic receptor family involved in behavior, cognitive function, mood and stress [45,46,47]. They are widespread in central nervous system [45,47,48], and when present presynaptically, they act as autoreceptors, and thereby inhibit the release of neurotransmitters [46]. Thus, the aim of the present study was to investigate the effects of an established low dose of BPA on sex differences in anxiety-like behaviors and the central catecholaminergic system in the adult mouse.

## 2. Results

### 2.1. Preliminary Analysis

Table 1 shows the descriptive statistics of the study. Preplanned comparisons were carried out to evaluate whether males and females within the same treatment group were different from each other. Cohen’s d shows the standardized difference of the effect, and thus indicates the overall effect size. Based on Cohen’s rules for defining the effect size, a *d* = 0.2 is regarded as small effect, *d* = 0.5 a medium effect and *d* =.8 a large effect [49]. 

### 2.2. Effects of Prenatal Exposure to BPA on Behaviors during the Free-Exploratory Open Field (FOF)

**Figure 1:**
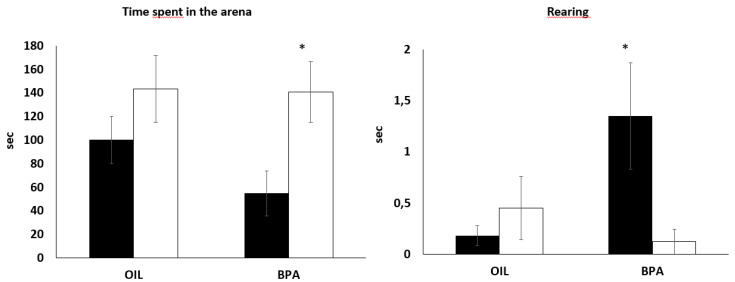
Effects of prenatal BPA exposure on behavioral responses of male and female mice in the free exploratory open field. (OIL: 14 males and 14 females; BPA: 14 males and 14 females). Dark columns = males. * *p* < 0.05 males vs. females BPA. Data are presented as mean ±SE.

In relation to the total time spent exploring the arena, independently of treatment, females explored more than males, F(1,52) = 7.49, *p* < 0.01, *ŋ_p_^2^* = 0.12 (Figure 1), but no main effect nor a sex dependent effect of BPA was found. However, our preliminary analysis with planned comparisons and effect size showed a large difference between males and females of the BPA group in relation to total exploration (*d* = 1.01), with the true effect size ranging from small to large (Table 1). No effects were found in relation to total risk assessment, while a significant interaction between BPA exposure and sex was found for total rearing, F(1,52) = 5.42, *p* < 0.05, *ŋ_p_^2^* = 0.09. BPA-exposed males performed more vertical exploration compared to BPA females (*p* = 0.04) (Figure 1).

### 2.3. Effects of Prenatal Exposure to BPA on Behaviors in the Elevated Plus Maze (EPM)

**Figure 2:**
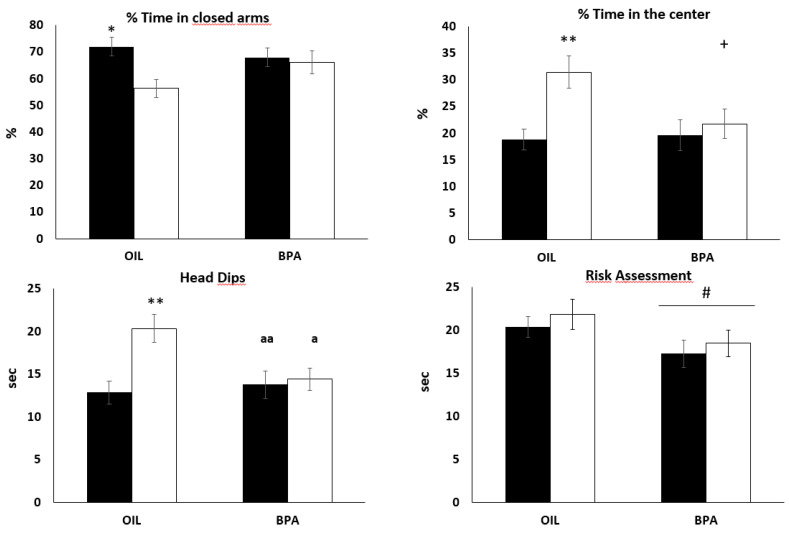
Effects of prenatal BPA exposure on behaviors in the elevated plus maze. (OIL: 14 males and 14 females; BPA: 14 males and 14 females); Dark columns = males. * *p* < 0.05, OIL males vs. OIL females; ** *p* < 0.01, OIL males vs. OIL females; ^+^ *p* = 0.06, OIL females vs. BPA females; **^a^** *p* < 0.05, OIL females vs. BPA females; ^aa^ *p* < 0.01, OIL females vs. BPA males; # *p* < 0.05 BPA group vs. OIL group. Data are presented as mean ± SE.

Independently of BPA exposure, females spent less time in the closed arms of the EPM compared to males, F(1,52) = 5,48, *p* =.02, *ŋ_p_^2^* = 0.09, while no main effect nor a sex dependent effect of BPA was found. Our exploratory analysis showed that males spent significantly more time in the closed arms compared to females (*d* = 1.21), with the true effect size ranging from medium to large, but this difference was not found in the BPA group (Figure 2). A similar result was found for the proportion of time spent in the center. Independently of BPA exposure, females spent more time in the center of the maze compared to males, F(1,52) = 7.54, *p* <.001, *ŋ_p_^2^* = 0.12. This main effect was qualified by a statistically significant interaction F(1,52) = 3.82, *p* =.05, *ŋ_p_^2^* = 0.06, showing that in the control group females spent significantly more time in the center compared to males (*p* < 0.01), while in the BPA-exposed group no sex difference was found. It is worth noting that females of the control group spent slightly more time in the center compared to females exposed to BPA (*p* = 0.06). A statistically significant main effect of treatment was found for total amount of risk assessment, F(1,52) = 4.44, *p* = 0.04, *ŋ_p_^2^* = 0.08, showing that BPA treated animals performed less risk assessment compared to controls (Figure 1).

In relation to exploratory behavior, independently of BPA exposure, females spent more time performing head dipping compared to males, F(1,52) = 7.49, *p* <.01, *ŋ_p_^2^* = 0.12. The extent of this sex difference depended on BPA exposure, F(1,52) = 5.31, *p* =.02, *ŋ_p_^2^* = 0.09. Specifically, while in the control group, females performed more head-dips compared to males (*p* < 0.001); no differences were found in the BPA-exposed groups. Moreover, the effect of BPA was such that males and females exposed to BPA performed significantly less head dipping compared to control females (*p* < 0.01 and *p* < 0.05 respectively) (Figure 1).

### 2.4. Effects of Prenatal Exposure to BPA on Reproductive Behaviors of Males

There were no statistically significant differences between BPA treated and control males in terms of latency to mount, *t*(18) = –0.87, *p* = 0.40 or latency to first intromission *t*(18) = −0.30, *p* = 0.76. These results indicate that prenatal exposure to this low dose of BPA did not cause long term effects in male reproductive behaviors.

### 2.5. Effects of Prenatal Exposure to BPA on Receptor Density and Affinity in the Locus Coeruleus

**Figure 3:**
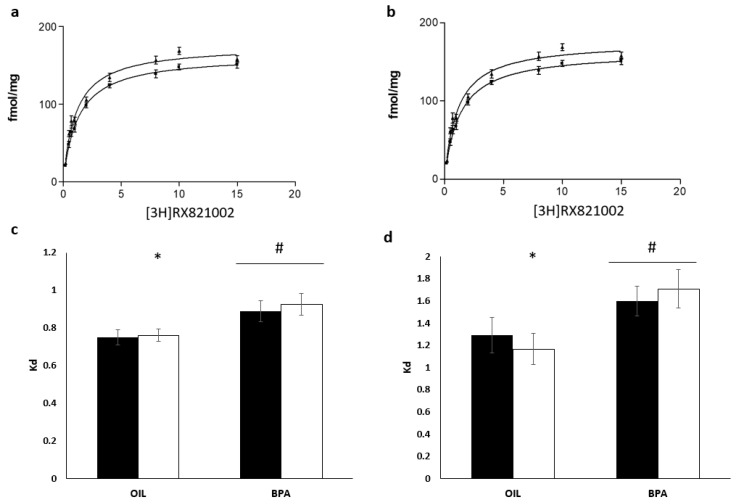
Effects of prenatal exposure to BPA on alpha_2_ adrenergic receptor affinity in the locus coeruleus and preoptic area. Binding curves for preoptic area of control (**a**) and BPA (**b**) groups. (**c**) Affinity (Kd) in the locus coeruleus; (**d**) affinity in the preoptic area. (OIL: eight males and eight females; BPA: eight males and seven females). Dark columns = males. * *p* < 0.05 OIL females vs. BPA females; # *p* < 0.05 BPA vs. OIL. Data are presented as mean ±SE. POA = preoptic area.

A two-way ANOVA showed no effects of treatment, sex nor interaction between treatment and sex on receptor density. Overall, BPA-exposed mice showed a lower affinity (*Kd*) compared to controls, F(1,28) =10.23, *p* < 0.01, *ŋ_p_^2^* = 0.26. An exploratory analysis performed using sex-stratified data showed that while males from the control group and BPA were not different from each other, control females had higher affinity compared to BPA-exposed females, *t*(14) = 2.53, *p* = 0.02 (Figure 3c).

### 2.6. Effects of Prenatal Exposure to BPA on Receptor Density and Affinity in the Medial Preoptic Area

Overall females had a slightly higher receptor density compared to males, independently of BPA exposure, F(1,27) = 3.87, *p* =.05, *ŋ_p_^2^* = 0.12 (Figure 3a). However, there were no statistically significant effects of treatment or of the interaction between treatment and sex. It is worth noting that our preliminary analyses showed that planned comparisons suggest that in the control group females had larger density of receptors compared to males (*d* = 1.08, Table 1) but such difference did not survive the cut off of p < 0.025 required by the planned comparison. As for the LC, BPA-exposed mice showed a lower affinity compared to controls, F (1,27) = 7.82, *p* < 0.01, *ŋ_p_^2^* = 0.22 (Figure 3d), but no main effect of sex or statistically significant interaction was found. However, a follow up exploratory analysis using sex-stratified data showed that females exposed to BPA had a significantly lower affinity compared to females from the control group, *t*(13) = 2.45, *p* = 0.03, while this was not found in the male group (Figure 3d).

## 3. Discussion

An important and new finding of the present work is that the affinity and density of alpha_2_-adrenergic receptors in the adult brain can be altered by prenatal exposure to BPA, implying the possibility that these receptors’ pharmacodynamics and expression can be organized by sex steroids early during development and that BPA may interfere with this developmental process. Although our analysis of sex differences on alpha_2_ adrenergic receptors pharmacodynamics does not allow us to make strong inferences, we found an effect of BPA on receptor affinity in female mice such that they had lower affinity compared to females of the control group. Moreover, prenatal exposure to BPA reduced receptor affinity in both males and females compared to controls. We could not find sex differences in terms of receptor density, although our preliminary analysis suggests that in the preoptic area of the control group, males might have lower density compared to females. The catecholaminergic system is very sensitive to the organizational and activational effects of gonadal hormones. Sex differences in the sizes and volumes of key brain regions for catecholaminergic functioning, such as the LC, the midbrain and the hypothalamus, and in the expression of TH and dopamine beta hydroxylase (DBH), have been reported in young and adult rodents [16,21,50,51,52,53]. Specifically for alpha_2_ adrenergic receptors, it is known that in the adult of rodent species, sex steroids can have both sex and region-specific effects on the binding characteristics [54] and on receptor density in the hypothalamus of the adult female [55].

This is not the first study showing the long term, organizational effects of a biological or social event early during development on alpha_2_ adrenergic receptors. Several studies showed that the affinity of alpha_2_ adrenergic receptors in several brain areas, such as the nucleus of the tractus solitarius and the hypothalamus, was changed by early postnatal exposure to oxytocin in male rats exposed to maternal caloric restriction [56] and that pups exposed to poor maternal care showed lower density of alpha_2_ adrenergic receptors in the LC, an effect that was associated with higher fearfulness [57]. However, how these long term effects are produced is not well understood. Several possibilities could be envisioned, although these are based on observations carried out in the adult animal. One requires a change in the conformation of the receptor, for example, through phosphorylation, an effect possibly mediated by estrogen [58]. Another possibility, however, is that, following early exposure to BPA, in the LC and mPOA a change in the expression of different populations of alpha_2_ adrenergic receptors occurs that would consequently influence the measured binding affinity. However, the antagonist [3H]RX821002 has similar affinity for the four alpha_2_ adrenergic receptors [59], suggesting that this may not be the case. Other mechanisms would require regulatory effects on alpha_2_ adrenergic gene expression, possibly mediated by estrogen receptors. At present, we cannot explain the mechanisms leading to the effects of BPA on affinity changes and further studies are required.

Activation of alpha_2_ adrenergic receptor in several areas of the hypothalamus is required for the expression of endocrine and proceptive behaviors in the female rodent [36,60,61,62,63] and regulates sex behaviors in males [28,29]. The reduced affinity reported in the mPOA of prenatally BPA-exposed mice points to a possible overall catecholaminergic overstimulation that may have important effects on the female’s estrous cycle, reproductive behavior, body thermoregulation and sleep-wake cycle [64]. Remarkably, prenatal exposure to a low, yet relevant dose of BPA did not alter adult male sexual behavior, a finding that disagrees with what has been previously reported in rats [42,43]. However, our null results are in line with Picot and colleagues [65] who reported that in male mice developmental exposure to BPA at TDI or NOAEL dose did not affect sexual behavior or alter their neuroanatomical organization of the preoptic area. However, it is interesting to point out that, in female rodents, studies on the effects of developmental BPA exposure on sexual behavior reported contrasting outcomes, depending on the specie/strain, method and length of exposure to the chemical [66,67]. Here we conducted tests on sexual behavior only on male mice, as female mice do not display complex patterns of sexual behaviors compared to male mice or to female rats [68,69,70]. It is possible, however, that female proceptive behaviors could be affected by BPA exposure and further studies might assess this possibility.

Acute activation of alpha_2_-adrenergic receptors in the adult animal has been associated with vasodepressor and anti-stress effects in the CNS, for which the alpha_2_ adrenergic receptors in the LC play a prominent role [45,71]. Early exposure to stress alters the number and affinity of alpha_2_ adrenergic receptors in the LC of adult animals [56,57]. Similarly to what we have observed in the mPOA, prenatal exposure to BPA decreased receptor affinity in the LC, suggesting that BPA-exposed mice, independently of sex, could be characterized by an elevated noradrenergic output that, behaviorally, should correlate with higher locomotor activity, more arousal and anxious-like behaviors, in a similar way to what occurs in chronically stressed adult mice. When tested on the EPM, BPA-exposed mice were less anxious (lower levels of risk assessment) than controls. In line with expectations, in the EPM, BPA-exposed male mice did not differ from BPA females, at odds with the behavioral sex differences observed in controls (male controls spent more time on the closed arms and less time in the center and explored by means of fewer head dips than females). Overall present results on emotional response to a novel environment in BPA mice are in line with the historical literature from our laboratory [9,26,27]. When adult mice were challenged in the FOF, we observed that BPA-exposed males spent less time exploring the novel compartment but performed more vertical exploration than BPA females, whereas no sex differences were observed in controls. Compared to previous studies (9,27,28), BPA effects on behavioral responses in the FOF and in the EPM were less pronounced here, probably due to the fact that maternal exposure was restricted at the last week of gestation and did not include the first postnatal period. We have indeed reported that a single, very low dose of BPA exposure induced behavioral changes in anxiety and exploration measures, particularly in response to postnatal exposure [27].

In a previous study [44], we reported that prenatal exposure to a single, very low dose of BPA in mice results in changes in the psychopharmacological profile of only one sex. Male mice showed no behavioral alterations due to the BPA prenatal exposure in an amphetamine-induced conditioning paradigm, whereas BPA-exposed females showed a disruption in the amphetamine-induced conditioning. These and the present results substantiate the idea that the developing catecholaminergic system is vulnerable to the disruptive effects of BPA during sensitive periods of development. They also underscore the possibility that females are more vulnerable to BPA exposure during critical periods of development, suggesting sex-specific alterations in the function of brain neurochemical systems involved in reward and arousal. It is important to emphasize that in the present work we did not use a multidose-response paradigm; instead we used an established, single low dose of BPA that has been showed to have important developmental effects on neurobiological and behavioral endpoints [26,27,41,44]. As a general consideration, our results confirm a consistent finding in current Endocrine Disrupting Chemicals (EDCs) research: exposure to low doses of BPA in utero may disrupt the development of sex-specific behaviors by altering normal steroid programming of the brain, and this disruption affects males and females differently [72]. This implies the relevance of including an analysis of both sexes and of sexually differentiated/dimorphic responses when assessing the effects of developmental exposure to EDCs.

## 4. Materials and Methods

Experiments were conducted in accordance with the European Communities

Council Directive of 24 November 1986 (86/EEC) and approved by the Italian Institute of Health.

### 4.1. Maternal Treatment

Adult female CD-1 mice (Mus musculus domesticus) born and reared in our lab but originally purchased from Charles River Italia (Calco, Lecco, Italy) were time mated and group housed with food (4RF21 Mucedola, Milano, Italy) and water ad libitum. New Polycarbonate cages and bottles were used in order to minimize exogenous sources of endocrine disruption, as it was reported that BPA leaching at environmental temperatures is minimal under such conditions [73]. Beginning on day 6 after detection of the vaginal plug, females were trained to spontaneously drink a small volume (0.05 mL) of corn oil from a modified syringe (without the needle and with a larger hole) introduced through the cage top every day after 6 h the light onset. This procedure allows for accurate chemical administration without the stress associated with gavage or injection [41]. Starting from gestation day (GD) 11, each female was housed alone in a cage (30 × 14 × 13 cm) and randomly assigned one of the following treatments (10–12 females/group): corn oil alone for the control group, 10 μg/kg body weight/day (*bw*/*d*) of BPA. From GD 11–18, each female drank 0.1 mL of corn oil per 50 g *bw*/*d*, with or without the chemicals.

### 4.2. Birth and Weaning

Within 12 h following parturition (postnatal day—PND1), each litter was culled to 10 pups (5 ± 1 males and 5 ± 1 females), which were returned to their biological mothers. The pups were weighted at PND 1, 3, 10,15, 20 and 40. On PND 25, offspring were weaned and mice were group-housed with same-sex littermates in propylene cages (41 × 24 × 14 cm^3^) till the moment they were used for testing. To avoid the animals receiving olfactory stimulus from the other sex, males and females were stabled in different rooms until the day of sacrifice. At adulthood (60 days old), one male and one female per litter underwent the EPM, FOF or a sexual behavior test. Behavioral tests were conducted between 15:00 and 17:00 in a testing room, where experimental mice were brought 3 h prior testing in order to habituate to the new conditions. One male and one female per litter were left undisturbed and used for brain analysis. The animals were maintained and tested under a normal 12 h light: 12 h dark cycle.

### 4.3. Free-Exploratory Open Field Test (FOF)

We used a FOF apparatus as described in detail in Palanza and coworkers [74]. Briefly, the apparatus consisted of two sections: a home-cage and an unfamiliar OF. In the OF, a lamp posed near the apparatus wall produced a bright zone and a dark one. A camera posed above apparatus videotaped the whole test. At the end of each observation, floor and walls were accurately cleaned up with water, alcohol and then water again. The day before testing, mice (*n* = 14) were individually housed in the home-cage section. The testing day, the home-cage section was put in the apparatus and the connecting door was opened allowing mouse entrance in OF. A cut-off of 10 min was used for those animals that did not enter the OF. These animals were included in the statistical analysis with a latency to enter the OF of 600 and 0 s spent in the OF, while being excluded from further behavioral analysis. After the first entry (with the four paws) into the OF, mice were given 5 min maximum time to explore the novel environment. Latency to enter the OF (the time from opening of the home cage to actual mouse entrance into the OF with all four paws), risk assessment (forward elongation of the head and shoulders occurring from the home cage while scanning the unfamiliar OF), walking, rearing (standing with the forepaws up) and time in the OF were scored by means of the specific software The Observer (Noldus, Wageningen, The Netherland) by a trained observer who was blind about exposure categories of the experimental subjects. In addition, by means of the Ethovision program (Noldus) latency, frequency and time spent in several OF zones (light, dark, corners, center, outer-ring, near the home cage, far from the home-cage) were determined along with distance traveled (cm), and speed of movement (velocity, cm/s).

### 4.4. Elevated Plus Maze

The day after the FOF test, one male and one female from each litter were tested on the EPM. Briefly, a mouse was removed from the home-cage and placed in the center of the apparatus for a 5 min test. A camera posed above apparatus videotaped the whole test. At the end of each observation, floor and walls were accurately cleaned up with water, alcohol and then water again. Conventional behaviors were scored by means of The Observer (Noldus). These comprised the frequencies of open and closed-arm entries (arm entry defined as all four paws into an arm) and total arm entries; and the amounts of time spent by the animals in open, central and closed parts of the maze. These data were used to additionally calculate percent open entries (open/total × 100) and percent time spent in different maze sections (location/300 × 100). In addition, frequency and duration of stretched attend postures (SAP; exploratory posture in which the mouse stretches its body forward without moving one or both hind paws), flat-back approach behavior (exploratory behavior where the mouse stretches to its full length while slowly moving forward) and risk assessment (stretched attend postures performed scanning the open arms with one or both hind paws still in the closed arm or central area) were measured and analyzed as total risk assessment behavior.

### 4.5. Sociosexual Behavior

When 90 days old, a sexual behavior test was performed. Experimental males were exposed to an unfamiliar (age and strain matched) female in estrous or proestrous state, while experimental females in estrus or proestrus states were exposed to unfamiliar (age and strain matched) males. Estrous stage was determined through vaginal smears and microscopic identification, according to [63]. Tests were conducted in a novel and unfamiliar transparent cage (27 × 20 × 13cm) with no bedding to allow for observation by the transparent cage floor. Two cameras were placed immediately above and below the cage, to allow videotape recording and the identification of all stages of socio-sexual behavior. Socio-sexual interaction was videotaped for a 30 min period and behavioral data were collected according to Dadomo and colleagues [75]. Data analysis was based on the following behavioral categories: (1) Exploration: sniffing the cage, sniffing with the nose up in the air, rearing, attention posture and digging. (2) Grooming, divided into self-grooming: wiping, licking, nibbling its own fur and scratching movements; and allo-grooming: when all those behaviors were directed to a female. (3) Affiliation: sniffing the female’s body, sniffing the female’s muzzle. (4) Sexual: anogenital sniffing, mounts and attempts to mount, genital self-grooming, intromission and ejaculation. For each behavior reported above, the latency to, the total duration and the number of times were also recorded. Tapes were analyzed and scored by a trained observer using specific software (The Observer, Noldus). Each male and female participated in the experiment only once.

### 4.6. Receptor Autoradiography and Quantification

When 60 days old, 16 males and 16 females (8 males and females from each group) were sacrificed. The brains were quickly removed and frozen in liquid nitrogen. Cryostat sections (10 µm) were cut at −18 °C, thaw-mounted onto gelatin-coated glass slides and placed under vacuum at 4 °C overnight. Saturation experiments were performed with 9 different concentrations of the antagonist [3H] RX821002 ((1,4-[6,7(n)3H]-benzodioxan-2-methoxy-2-yl)-2-imidazole hydrochloride; specific activity 60.0 Ci/mmol; Amersham). Sections were incubated with [3H]RX821002 in buffer (50 mM K2HPO4/KH2PO4, ph 7,4; 5 mM MgCl2, 10 microM pargyline) for 60 min at 37 °C; washed in buffer twice for 2 min on ice, in distilled water (30s on ice); and dried under a stream of cold air. Non-specific binding was determined in the presence of a 1000-fold excess of yohimbine. Quantification of [3H]RX821002 binding sites was performed by autoradiography. Brain sections were exposed along with 20 µm 3H-microscale standards on tritium-sensitive Hyperfilm-3H (Amersham, Braunschweig, Germany) for 10 weeks at a temperature of 8–10 °C.

When 60 days old, 16 males and 16 females (8 males and females from each group) were sacrificed. The brains were quickly removed and frozen in liquid nitrogen. Cryostat sections (10 µm) were cut at −18 °C, thaw-mounted onto gelatin-coated glass slides and placed under vacuum at 4 °C overnight. Saturation experiments were performed with 9 different concentrations of the antagonist [3H] RX821002 ((1,4-[6,7(n)3H]-benzodioxan-2-methoxy-2-yl)-2-imidazole hydrochloride; specific activity 60.0 Ci/mmol; Amersham). Sections were incubated with [3H]RX821002 in buffer (50 mM K2HPO4/KH2PO4, ph 7,4; 5 mM MgCl2, 10 microM pargyline) for 60 min at 37 °C; washed in buffer twice for 2 min on ice, in distilled water (30s on ice); and dried under a stream of cold air. Non-specific binding was determined in the presence of a 1000-fold excess of yohimbine. Quantification of [3H]RX821002 binding sites was performed by autoradiography. Brain sections were exposed along with 20 µm 3H-microscale standards on tritium-sensitive Hyperfilm-3H (Amersham, Braunschweig, Germany) for 10 weeks at a temperature of 8–10 °C.

**Figure 4:**
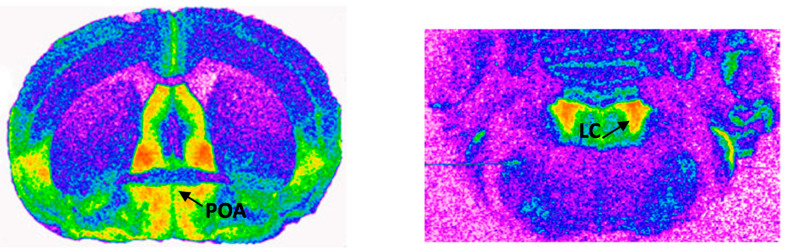
Autoradiograms showing the distributions of alpha_2_-adrenergic receptors in the preoptic area (POA, **left**) and locus coeruleus (LC, **right**). Red = higher density of alph2-adrenergic receptors.

The films were analyzed densitometrically with a computerized image analysis system (MCID AIS, Imaging Research Inc., St Catherines, ON, Canada). Gray values of the standards were used to determine the amount of radioactivity bound to tissue sections, which was expressed in phentomoles bound per milligram (fmol/mg) tissue equivalents. The regions analyzed (Figure 4) was anatomically localized with a mouse brain atlas [76]. Data from saturation and competition experiments were generated with a curve fitting program: Graph Pad Prism (Graph Pad Software Inc., San Diego, CA, USA).

### 4.7. Statistical Analysis

For each dependent variable we present means and standard deviation for each sex across treatment in Table 1. Preliminary statistical analyses to explore sex differences across treatments (independent of treatment effect) were carried out by means of planned comparisons with Bonferroni’s adjustment (α_PC_ = 0.025). Cohen’ d effect size and its relative 95% CI are also presented. The possibility that BPA exposure could affect sex differences was investigated by means of 2 × 2 factorial ANOVA and Tukey’s HSD was used for post-hoc multiple comparisons. Following Rich-Edwards et al. [77], in case of a statistically non-significant interaction, differential main effects of treatment were investigated by sex stratifying the data. Specifically, we investigated the possible effects of prenatal exposure to BPA separately for each sex by means of independent t-tests. All statistical analyses were performed using the SPSS 26 (IBM statistics, Armonk, NY, USA).

## Figures and Tables

**Table 1:** Descriptive statistics for each dependent variable in relation to sex and treatment.

	OIL					BPA				
	Females		Males			Females		Males		
Outcome	M	SD	M	SD	*d*	M	SD	M	Sd	*d*
**FOF**										
Exp	143.10	105.78	100.05	74.48	0.46 [−0.27; 1.21]	140.81	96.81	54.73	70.98	1.01 [0.22; 1.79] *
RA	35.5	27.63	33.9	31.18	0.46 [−0.27; 1.21]	27.92	31.03	19.93	17.14	0.43 [−0.31; 1.17]
Rear	0.45	1.19	0.18	0.41	0.29 [−0.43; 1.02]	0.12	0.48	1.35	1.96	0.85 [0.08; 1.61]
**EPM**										
%CA	56.37	12.52	71.96	13.03	1.21 [0.41; 2.02]*	66.10	16.43	68.00	13.55	0.12 [−0.84; 0.60]
%CE	31.45	11.23	18.84	7.14	1.33 [0.53; 2.14]*	21.77	10.44	19.65	10.78	0.20 [−0.52; 0.92]
HD	20.35	6.23	12.85	5.03	1.32 [0.52; 2.12]*	14.42	4.92	13.78	5.95	0.11 [−0.60; 0.84]
RA	21.87	6.66	20.42	4.53	0.25 [−0.55; 1.05]	18.5	5.74	17.28	5.92	0.20 [−0.51; 0.93]
**POA**										
Density	177.81	15.92	161.97	13.25	1.08 [0.04; 2.11]	182.40	19.42	174.87	17.02	0.40 [−0.53; 1.34]
Kd	1.65	0.40	1.29	0.45	0.29 [−1.23; 0.65]	1.70	0.46	1.59	0.37	0.26 [−0.73; 1.26]
**LC**										
Density	195.72	7.99	190.06	9.99	0.62 [−0.37; 1.62]	195.00	14.61	192.88	9.43	0.17 [−0.81; 1.15]
Kd	0.76	0.9	0.74	0.11	0.10 [−0.87; 1.08]	0.92	0.15	0.88	0.15	0.23 [−0.47; 1.21]

Note. FOF = free exploratory open field; Exp = total exploration time (s); RA = total risk assessment (s); Rear = total rearing (s); EPM = elevated Plus Maze; %CA = % time in closed arms; %CE = % time in center; HS = total number of head dips; RA = total time in risk assessment; POA = preoptic area; LC = locus coeruleus; d = Cohen’s *d*, presented with 95% confidence interval; * *p* < 0.025 (preplanned comparisons with Bonferroni’s adjustment).
